# SDANN May Be Used as a Screening Factor of Obstructive Sleep Apnea in Adult Individuals

**DOI:** 10.1155/bmri/3813278

**Published:** 2026-08-27

**Authors:** Nuntigar Sonsuwan, Chayapa Charoenwaree, Nipon Chattipakorn, Kittisak Sawanyawisuth

**Affiliations:** ^1^ Department of Otolaryngology, Faculty of Medicine, Chiang Mai University, Chiang Mai, Thailand, cmu.ac.th; ^2^ Jainad Narendra Hospital, Chai Nat, Thailand; ^3^ Cardiac Electrophysiology Research and Training Center, Faculty of Medicine, Chiang Mai University, Chiang Mai, Thailand, cmu.ac.th; ^4^ Department of Medicine, Faculty of Medicine, Khon Kaen University, Khon Kaen, Thailand, kku.ac.th

**Keywords:** heart rate variability, Holter, obstructive sleep apnea, ROC, sensitivity, specificity

## Abstract

**Background:**

Obstructive sleep apnea (OSA) is a common disease and can be diagnosed with polysomnography (PSG). However, it is limited and has a long waiting time. This study is aimed at evaluating if HRV can be a screening tool for OSA in a real‐world setting.

**Methods:**

This was a prospective study that enrolled adult patients aged 18 years or older who were suspected of OSA and underwent both PSG and HRV tests. A stepwise multivariable logistic regression analysis was executed to identify predictors of OSA using HRV parameters.

**Results:**

There were 15 patients with simple snoring and 15 patients with OSA who participated in the study. Among the six parameters of HRV, four parameters were included in the predictive model for OSA. Of those, only the standard deviation of the average of NN intervals in all 5‐min segments of the entire recording (SDANN) was independently associated with OSA, with an adjusted odds ratio of 0.951 (95% confidence interval of 0.905, 0.999). The area under the receiver operating characteristic curve of SDANN for OSA was 77.11%. An SDANN cut point of less than 106 ms had a sensitivity of 80.00% and a specificity of 60.00% for identifying OSA.

**Conclusions:**

The HRV test may be used as a screening tool for OSA in adult individuals suspected of OSA. A low SDANN may be an indicator of OSA, with a sensitivity of 80.00%.

## 1. Introduction

Obstructive sleep apnea (OSA) is a common disease and causes a large economic burden [1]. A study from Europe showed that the estimated OSA prevalence was 18% and that the disease had an economic burden of 184 billion euros. Even though OSA is highly prevalent, at least 80% of patients with OSA may be undiagnosed due to a lack of suggestive symptoms or an underestimation of symptoms [2, 3]. Polysomnography (PSG), the gold standard for OSA diagnosis, is limited and has a long waiting time due to limited availability and a large volume of suspected patients [4]. Alternative devices to diagnose OSA may be needed.

Currently, clinical questionnaires such as the Epworth Sleepiness Scale (ESS) and the STOP‐BANG questionnaire, alongside home sleep apnea testing (HSAT), are widely utilized as standard screening methods prior to referring a patient for comprehensive in‐laboratory PSG [5–7]. Although these tools are heavily relied upon, they have distinct limitations. Questionnaire‐based assessments depend on subjective self‐reporting and often require the presence of a bed partner to confirm symptoms, making them susceptible to recall and reporting biases. HSAT provides objective data and is more accessible than in‐lab PSG, but it still requires specialized equipment, relies on patient compliance for proper sensor placement at home, and can be subject to technical failures or data loss. In contrast, analyzing heart rate variability (HRV) derived from routine Holter ECG offers several potential advantages over these conventional screening methods. First, HRV provides a completely objective physiological measure of autonomic nervous system fluctuations during sleep, eliminating the subjectivity associated with questionnaires. Furthermore, because ambulatory Holter ECG is already a widely accessible and standard diagnostic tool used to evaluate cardiovascular conditions—a patient population with a notoriously high prevalence of undiagnosed OSA—extracting HRV parameters from these recordings enables opportunistic screening. This approach could allow clinicians to identify high‐risk OSA patients seamlessly during routine cardiac evaluations, without the immediate need for additional specialized sleep testing equipment.

Additionally, recent advancements in wearable technology and single‐lead ECG analysis have introduced novel screening methodologies for OSA. For instance, several studies have successfully utilized machine‐learning models to analyze single‐lead ECG data, detecting the presence of apnea or hypopnea events on an epoch‐by‐epoch (e.g., 1‐min) basis. These models subsequently derive an estimated AHI to evaluate diagnostic sensitivity and specificity [8–10]. Although these machine‐learning–based approaches demonstrate high accuracy, standard HRV analysis was selected for the present study due to several distinct practical advantages. First, conventional HRV parameters are mathematically straightforward, computationally efficient, and avoid the “black box” nature of complex neural networks, which typically require extensive training datasets and proprietary software. Second, standard HRV metrics are already universally integrated and automatically generated by virtually all commercial Holter ECG systems currently used in clinical practice. This standardized, built‐in availability allows our findings to be immediately translated into existing clinical workflows, offering a highly accessible method for opportunistic OSA screening without the need for specialized algorithmic processing.

Although apneic events occur exclusively during sleep, the physiological consequences of OSA—most notably chronic sympathetic overactivity, altered baroreflex sensitivity, and systemic autonomic dysfunction—are known to persist throughout daytime wakefulness [11, 12]. Consequently, evaluating HRV exclusively during the nocturnal period may only capture acute autonomic responses to apneic episodes, missing the comprehensive autonomic dysregulation associated with the disease. Measuring 24‐h HRV provides a broader reflection of this cumulative, day‐and‐night autonomic burden. Furthermore, from a practical standpoint, 24‐h Holter monitoring is the universal standard for ambulatory electrocardiography. Time‐domain metrics such as SDANN (standard deviation of the average NN intervals) are specifically established and standardized based on 24‐h recordings [13]. Leveraging the full 24‐h recording therefore allows for seamless, opportunistic screening of OSA in patients already undergoing routine cardiovascular evaluation, without the complexity of accurately isolating the nocturnal sleep window.

HRV is a medical test to evaluate autonomic dysfunction [14, 15]. A previous study found that patients with severe OSA had a significantly higher percentage of low frequency (LF) than the nonsevere OSA group in nonrapid eye movement sleep stage (44.1% vs. 41.9%; *p* < 0.001) [16, 17]. Additionally, HRV has been shown to be a screening tool for OSA by machine learning [4, 18]. Its sensitivity and specificity for OSA diagnosis were 76%–79% and 75%–92%, respectively [4, 18]. Although previous investigations have established a foundational link between altered HRV parameters and OSA, these studies possess notable limitations that hinder their immediate clinical translation. Many prior HRV studies relied on relatively small, highly controlled patient cohorts or sanitized, publicly available datasets. Furthermore, they often excluded patients with concurrent cardiovascular comorbidities, thereby failing to capture the physiological complexity, artifacts, and “noise” inherent in everyday clinical environments. Consequently, a significant research gap remains: There is a critical need to validate the utility of HRV metrics as an OSA screening tool using unselected, real‐world clinical data. The present study directly addresses this gap. By utilizing a clinical cohort drawn from routine practice—complete with the typical variations in age, BMI, and comorbidities—we aim to evaluate whether standard Holter‐derived HRV parameters, specifically SDANN, can maintain their diagnostic performance and serve as a practical, reliable screening tool for OSA in real‐world clinical settings. This study is aimed at evaluating if HRV can be a screening tool for OSA in a real‐world setting.

## 2. Materials and Methods

This was a prospective, analytical study conducted at the Department of Otolaryngology, Faculty of Medicine, Chiang Mai University, Thailand. The inclusion criteria were an age of 18 years or older, suspected OSA, and completion of diagnostic PSG and 24‐h ambulatory Holter monitoring, from which HRV parameters were subsequently derived. The exclusion criteria were pregnant women and patients with a history or evidence of any malignant arrhythmia, including atrial fibrillation, atrial flutter, an atrioventricular conduction defect, frequent ectopic beats, or extreme sinus arrhythmia. Eligible patients were enrolled consecutively. The study protocol was approved by the Research Ethics Committee, Chiang Mai University, Thailand (413/2562). A written informed consent was provided by each study participant prior to study participation.

Eligible patients were evaluated for baseline characteristics, physical signs, PSG, and HRV testing. Baseline characteristics included age, sex, and comorbid diseases, whereas physical signs included body weight, height, body mass index, and oropharyngeal examination findings: macroglossia, soft palate flutter, Friedman tongue position, and tonsillar grade. An overnight PSG was performed for at least 6 h for one night, yielding results for AHI, lowest oxygenation, and average oxygenation. The initiation of both the PSG and the 24‐h HRV recording occurred on the same calendar day. Specifically, the HRV monitoring began at 4:00 PM using the GE SEER Light Extend device, and the PSG (using Somnocheck v2.04) commenced later that evening at 8:00 PM. The HRV recording continued until 4:00 PM the following day, capturing the entire sleep period and the subsequent 24 h. HRV was assessed using both time‐ and frequency‐domain parameters. Prior to parameter calculation, the 24‐h ECG data were preprocessed by filtering out noises, ectopic beats and artifacts, and maintaining only valid normal‐to‐normal (NN) intervals. Following preprocessing, HRV analysis was conducted using the MARS Ambulatory ECG analysis system. For each patient, a single global 24‐h value was calculated for each HRV parameter. The analysis consisted of both time‐ and frequency‐domain results. The time‐domain results comprised three parameters, all recorded in milliseconds (ms): the standard deviation of all NN intervals (SDNN), SDANN, and the square root of the mean of the sum of the squares of differences between adjacent NN intervals (rMSSD). The frequency‐domain results also comprised three parameters: LF (0.04–0.15 Hz), high frequency (HF; 0.15–0.40 Hz), and the LF/HF ratio.

The statistical analyses were composed of two parts: baseline data comparison between groups and predictive model for OSA. Patients were categorized into two groups: primary snoring and OSA. Descriptive statistics were used to calculate median (range) and number (percentage) for numerical and categorical variables, respectively. Inferential statistics were used to compare the differences between both groups. Wilcoxon rank sum test was used to compare the differences between both groups for numerical variables, whereas Fisher′s exact test was used to compare the differences between two proportions.

Predictors of OSA were executed by stepwise method of multivariable logistic regression analysis by using six HRV parameters including SDNN, SDANN, rMSSD, LF, HF, and LF/HF ratio. Studied variables were computed for a *p* value by univariable logistic regression analysis. These six HRV parameters were included in the stepwise, multivariable logistic regression analysis. Factors retaining in the predictive model were those with a *p* value by multivariable step of less than 0.25. The model was tested for a goodness of fit by the Hosmer–Lemeshow method. A *p* alue by the Hosmer–Lemeshow chi square of more than 0.05 indicated a goodness of fit. A numerical predictor for being OSA was computed for appropriate diagnostic cut point by a receiver operating characteristic (ROC) curve. Sensitivity and specificity for the best cutoff point for OSA diagnosis were reported. All statistical analyses were computed by using STATA software Version 18.5 (College Station, Texas, United States). We followed the methods of Limsirorat et al. [19].

## 3. Results

There were 15 patients with simple snoring and 15 patients with OSA who participated in the study. Table 1 showed baseline characteristics of both groups. Four factors were significantly different between the simple snoring group and OSA group: body weight, height, body mass index, and presence of macroglossia. The OSA group had a significantly higher body mass index (36.31 vs. 22.89 kg/m^2^; *p* < 0.001) and higher proportion of macroglossia (40.00% vs, 0; *p* = 0.017) than the simple snoring group.

**Table 1:** Baseline characteristics and physical signs of patients who underwent heart rate variability test categorized by either simple snoring or obstructive sleep apnea (OSA).

Factors	Simple snoring *n* = 15	OSA *n* = 15	*p*
Age, years	51 (21–85)	32 (23–66)	0.182
Male sex	6 (40.00)	7 (50.00)	0.715
Comorbid diseases			0.931
Hypertension	4 (26.67)	9 (60.00)	0.139
Diabetes mellitus	1 (6.67)	2 (13.33)	0.999
Dyslipidemia	1 (6.67)	6 (40.00)	0.080
Stroke	1 (6.67)	0	0.999
Physical signs			
Body weight, kg	60 (45–87)	89 (68–145)	< 0.001
Height, cm	162 (150–175)	170 (150–195)	0.049
Body mass index, kg/m^2^	22.89 (20.00–33.15)	36.31 (23.52–44.18)	< 0.001
Oropharyngeal examination			
Macroglossia	0	6 (40.00)	0.017
Flutter soft palate	5 (33.33)	4 (26.67)	0.999
Friedman tongue position			0.130
1	5 (33.33)	2 (15.38)	
2	8 (53.33)	4 (30.77)	
3	2 (13.33)	6 (46.15)	
4	0	1 (7.69)	
Tonsillar grading			0.226
1	0	1 (7.69)	
2	11 (73.33)	6 (46.15)	
3	4 (26.67)	5 (38.46)	
4	0	1 (7.69)	

*Note:* Data are presented as median (range) or numbers (proportion).

For PSG and HRV results, the simple snoring group and OSA group had significantly different factors of PSG and time domain parameters of HRV including AHI, lowest oxygenation, average oxygenation, SDNN, SDANN, and rMSSD (Table 2). The OSA group had shorter SDNN (110 vs. 134 m; *p* = 0.003), SDANN (99 vs. 124 ms; *p* = 0.011), and rMSSD (24 vs. 33 ms; *p* = 0.013) than the simple snoring group. There was no significantly different frequency domain parameters of HRV between both groups. Note that these HRV parameters were extracted from the same 24‐h recording.

**Table 2:** Baseline characteristics of patients who underwent heart rate variability test categorized by either simple snoring or obstructive sleep apnea (OSA).

Factors	Simple snoring *n* = 15	OSA *n* = 15	*p*
Polysomnography			
AHI, times/h	1.5 (0.1–4.8)	37 (7–62)	< 0.001
Lowest oxygenation, %	85 (67–91)	78 (44–92)	0.004
Average oxygenation, %	96.6 (95.0–98.1)	94.5 (79.0–97.9)	0.004
Heart rate variability			
Time domain parameters			
SDNN, ms	134 (74–191)	110 (74–150)	0.003
SDANN, ms	124 (63–179)	99 (68–123)	0.011
rMSSD, ms	33 (20–57)	24 (1.5–45)	0.013
Frequency domain parameters			
LF, ms	20.10 (11.96–38.98)	17.33 (7.63–30.64)	0.141
HF, ms	14.25 (6.65–28.37)	8.81 (5.28–19.41)	0.054
LF/HF ratio	1.37 (0.82–2.54)	1.65 (0.95–2.74)	0.281

*Note:* Data are presented as median (range).

Abbreviations: HF, high frequency; LF, low frequency; rMSSD, square root of the mean of the sum of the squares of differences between adjacent NN intervals; SDANN, standard deviation of the average of NN intervals in all 5‐min segments of entire recording; SDNN, standard deviation of all NN intervals.

**Figure 1:**
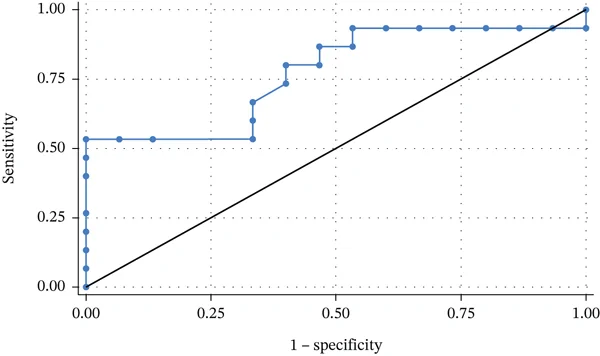
The receiver operating characteristic (ROC) curve by standard deviation of the average of NN interval in all 5‐min segments of entire recording (SDANN) for obstructive sleep apnea (OSA). Area under the ROC curve was 77.11% (95% confidence interval of 59.39%, 94.82%).

Among six parameters of HRV, four parameters were in the predictive model for OSA including SDANN, rMSSD, LF, and LF/HF ratio (Table 3). Of those, only SDANN was independently associated with OSA with an adjusted odds ratio of 0.951 (95% confidence interval of 0.905, 0.999). The Hosmer–Lemeshow chi square of the model was 5.85; *p* = 0.664 indicating goodness of fit of the model. The area under ROC curve of SDANN for OSA was 77.11% (95% confidence interval of 59.39%, 94.82%) as shown in Figure 1. An SDANN cut point of less than 106 ms had sensitivity of 80.00% and specificity of 60.00% for being OSA.

**Table 3:** Factors associated with presence of obstructive sleep apnea in patients who underwent heart rate variability test by stepwise logistic regression analysis.

Factors	Unadjusted odds ratio (95% confidence interval)	Adjusted odds ratio (95% confidence interval)
SDNN	0.951 (0.913, 0.989); 0.014	Not included
SDANN	0.952 (0.914, 0.991); 0.017	0.951 (0.905, 0.999); 0.048
rMSSD	0.904 (0.831, 0.984); 0.020	0.802 (0.622, 1.035); 0.090
LF	0.927 (0.844, 1.019); 0.119	1.183 (0.912, 1.534); 0.205
HF	0.853 (0.729, 0.998); 0.048	Not included
LF/HF ratio	2.358 (0.426, 13.051); 0.326	0.096 (0.002, 3.657); 0.208

*Note:* Factors included in the model were parameters of the heart rate variability.

Abbreviations: HF, high frequency; LF, low frequency; rMSSD, square root of the mean of the sum of the squares of differences between adjacent NN intervals; SDANN, standard deviation of the average of NN intervals in all 5‐min segments of entire recording; SDNN, standard deviation of all normal‐to‐normal intervals.

## 4. Discussion

This study showed that a particular HRV parameter can be a potential screening and diagnostic factor for OSA:SDANN. Even though SDNN, rMSSD, and HF parameters were significant by univariable logistic regression analysis (Table 3, second column), these parameters did not retain or become significant by multivariable logistic regression analysis (Table 3, third column). These findings may indicate that only SDANN was a significant factor after adjusting for other HRV parameters.

A recent systematic review of 12 included studies with 513 patients with OSA and 340 controls showed variable results of HRV parameters between both groups [20]. For example, LF was significantly higher in patients with OSA than controls in six studies, four nonsignificant results on LF, and one study had the reverse findings. For SDANN, there were seven studies evaluating it between patients with OSA and controls. Of those, two studies found that patients with OSA and controls had comparable SDANN [21, 22]; one study found that patients with OSA had shorter SDANN than controls significantly (108.63 vs. 126.95 ms: *p* < 0.05) [23]. This study found similar findings with the previous studies that SDANN in patients with OSA was significantly shorter than controls or patients with simple snoring (Table 2). The SDANN parameters in patients with severe OSA were also comparable with the previous study: 99 versus 108.63 ms [23]. Unlike this study, the previous studies did not adjust the HRV parameters with the logistic regression analysis. This study performed an adjustment among the HRV parameters and found that only SDANN was a significant predictor of OSA compared with controls.

Using SDANN as a predictor of OSA, the sensitivity was 80.00%. These results may imply that the HRV test with SDANN less than 106 ms may be suggestive for OSA. The sensitivity of SDANN may be comparable with other screening tests such as STOP‐BANG or Berlin questionnaire. Both screening tools had sensitivity of 81.46% and 86.42%, respectively [24]. Regarding specificity, the SDANN had comparable specificity with Berlin questionnaire (60.00% vs. 52.94%) but quite lower than STOP‐BANG questionnaire at 82.35% [24]. However, high sensitivity for OSA screening may be justified in this setting as early detection to prevent future cardiovascular consequences from OSA [25]. As HRV test may be more convenient and had shorter waiting time, low SDANN may be used as a screening diagnostic parameter for OSA. It is also important to note that the specificity of STOP‐BANG in that context is superior to the specificity of SDANN observed in the present study. However, comparing an objective physiological marker to a questionnaire highlights distinct clinical advantages for SDANN. Questionnaire‐based assessments like STOP‐BANG are inherently subjective; they rely on patient reporting, recall, and often require the presence of a bed partner to confirm symptoms like loud snoring or witnessed apneas. Consequently, they may be less reliable in patients who live alone or those who underreport their symptoms. In contrast, SDANN offers an objective, automated physiological metric that does not depend on patient literacy, memory, or bed partner observations. Furthermore, a unique clinical advantage of SDANN is its potential for opportunistic screening. Because SDANN can be extracted from standard ambulatory ECG (Holter) recordings, it allows clinicians to screen for OSA in patients undergoing routine cardiovascular evaluations without requiring an additional questionnaire or screening step. Therefore, rather than replacing highly effective questionnaires like STOP‐BANG, SDANN may serve as an invaluable, objective complement—particularly in solitary patients or those already undergoing cardiac monitoring.

The OSA group in this study had the median AHI of 37 times/h or categorized in the severe OSA. These findings may indicate the skewed distribution of our study population, with a predominant number of patients classified as having severe OSA. This skew reflects the referral nature of our specialized sleep clinic, where patients typically present with significant symptoms and a high pretest probability of the disease. Consequently, the diagnostic performance of SDANN observed in this study may be overestimated when compared with a general, unselected population. The results could differ significantly if a larger proportion of mild‐to‐moderate OSA patients were included. Because a broad screening tool must be effective across all severities of a disease, the current findings do not fully support the use of SDANN for general population screening. Instead, our results suggest that SDANN may be utilized as a preliminary case‐finding or triage tool in high‐risk clinical settings. Future prospective studies involving community‐based, unselected cohorts are necessary to validate the utility of SDANN as a true screening tool across the full spectrum of OSA severity.

There are some limitations in this study. First, the sample size in this study was small. However, SDANN still declared statistical significance. We also traced back the power of SDANN and found that the power was 87.5%. Further larger studies may be needed to confirm the results of this study as well as more patients with mild or moderate OSA. Second, the predictive model for OSA comprised only HRV parameters. Potential confounding factors were not fully controlled for in the primary analysis. In particular, BMI and macroglossia differed significantly between groups and may have influenced the association between SDANN and OSA. Although we explored models additionally adjusting for these variables, the estimates were unstable, likely due to the limited sample size. Therefore, residual confounding cannot be excluded. Finally, the control group in this study was patients with primary snoring, not healthy control participants.

## 5. Conclusions

HRV test may be used as a screening tool for OSA in adult individuals suspected of OSA. Low SDANN may be an indicator for OSA with sensitivity of 80.00%.

## Author Contributions

All authors contributed to the study conception, data collection, or data interpretation. Statistical analysis was performed by Kittisak Sawanyawisuth. The first draft of the manuscript was written by Nuntigar Sonsuwan, and other authors commented and modified the manuscript.

## Funding

The authors have nothing to report.

## Disclosure

All authors read and approved the final manuscript.

## Conflicts of Interest

The authors declare no conflicts of interest.

## Acknowledgments

This study was partially supported by The National Research Council of Thailand (N42A690147 to NC) and the Chiang Mai University Center of Excellence Award (NC).

## Data Availability

The data that support the findings of this study are available on request from the corresponding author. The data are not publicly available due to privacy or ethical restrictions.
